# Pharmacokinetics of isoniazid, rifampicin, pyrazinamide and ethambutol in Indian children

**DOI:** 10.1186/s12879-015-0862-7

**Published:** 2015-03-14

**Authors:** Aparna Mukherjee, Thirumurthy Velpandian, Mohit Singla, Kunwar Kanhiya, Sushil K Kabra, Rakesh Lodha

**Affiliations:** Department of Pediatrics, All India Institute of Medical Sciences, New Delhi, India; Department of Ocular Pharmacology, All India Institute of Medical Sciences, New Delhi, India

**Keywords:** Tuberculosis, Children, Plasma concentrations, Anti-tubercular therapy, Malnutrition

## Abstract

**Background:**

The available pharmacokinetic data on anti-tubercular drugs in children raises the concern of suboptimal plasma concentrations attained when doses extrapolated from adult studies are used. Also, there is lack of consensus regarding the effect of malnutrition on pharmacokinetics of anti-tubercular drugs in children. We conducted this study with the aims of determining the plasma concentrations of isoniazid, rifampicin, pyrazinamide and ethambutol achieved with different dosage of the anti-tubercular drugs so as to provide supportive evidence to the revised dosages and to evaluate the effects of malnutrition on the pharmacokinetics of these drugs in children. We also attempted to correlate the plasma concentrations of these drugs with clinical outcome of therapy.

**Method:**

Prospective drug estimation study was conducted in two groups of children, age 6 months to 15 years, with tuberculosis, with or without severe malnutrition, receiving different dosage of daily anti- tubercular therapy. The dosage (range) of isoniazid was 5 (4-6) and 10 (7-15) mg/kg in the two groups, respectively, that of rifampicin-10 (8–12) and 15 (10–12) mg/kg, respectively, both the groups received same dose of pyrazinamide (30–35 mg/kg) and ethambutol (20–25 mg/kg). All four drugs were simultaneously estimated by liquid chromatography-mass spectrometry (LC-MS/MS).

**Results and conclusion:**

The median (IQR) C_max_ of isoniazid increased significantly from 0.6 (0.3,1.2) μg/mL to 3.4 (1.8, 5.0) μg/mL with increase in the dose. Plasma rifampicin concentrations increased only marginally on increasing the dose [median (IQR) C_max_: 10.4 (7.2, 13.9) μg/mL vs. 12.0 (6.1, 24.3) μg/mL, p = 0.08]. For ethambutol, 55.9% of the children had inadequate 2-hour concentrations. Two-hour plasma concentrations of at least one drug were low in 59 (92.2%) and 54 (85.7%) children in the two dosing regimen, respectively. We did not observe any effect of malnutrition on pharmacokinetic parameters of the drugs studied. We did not observe an association between low plasma drug concentrations and poor outcome. We may have to be cautious while increasing the doses and strive to asses other factors influencing the drug concentrations and treatment outcomes in children.

**Electronic supplementary material:**

The online version of this article (doi:10.1186/s12879-015-0862-7) contains supplementary material, which is available to authorized users.

## Background

Escalating reports of treatment failure and emergence of resistance worldwide with the use of standard short course chemotherapy for tuberculosis (TB) reflect a worrying trend. In 2013, 20% of those previously treated for tuberculosis had multidrug resistance [1]. Sub-therapeutic plasma concentrations of anti-tubercular drugs in adults have been implicated as one of the causes of treatment failure and emergence of resistance [2-5].

The pharmacokinetic data on anti-tubercular drugs in children raises the concern of suboptimal plasma concentrations attained when the same mg/kg dose used in adults is extrapolated to children [6-11]. Children absorb, metabolize and eliminate these drugs in a different manner than adults. It is extrapolation of adult doses to children, without correctly accounting for the changes in pharmacokinetics with growth and maturation, etc. that may lead to incorrect dosing in children. Though isoniazid has been studied extensively, there are relatively fewer studies on rifampicin, pyrazinamide and ethambutol in children. These studies vary in the type of assay used for measurement of drug concentrations, sampling schedule adopted, type of anti-tubercular therapy (ATT) administered– intermittent or daily and type of formulations used–fixed dose combination or separate single formulations. These variations make direct comparison of data obtained from the available pharmacokinetic studies in children challenging.

After a review of available data, WHO has recommended revision of the doses of isoniazid, rifampicin and pyrazinamide in children, with an intent to reach the usually recommended targets for adults [12].

Malnutrition is associated with various processes in the body which may influence the pharmacokinetics of the anti-tubercular drugs - malabsorption, as well as enteric infections, deranged drug metabolizing enzymes, alteration in the protein binding of drugs, an increased or decreased renal clearance, and changes in the body composition (lower fat and lean body mass) [13,14]. If patients absorb only portions of their drug regimen, effectively they are exposed to a regimen of fewer anti-tubercular drugs or even monotherapy leading to the selection of resistant strains [13-15]. The effect of malnutrition on pharmacokinetics of anti-tubercular drugs in children has not been very well explored. The few studies which are available lack consensus. In a recently published study, Ramachandran et al. reported on the pharmacokinetics of three anti-tubercular drugs in Indian children using intermittent regimen and observed that the drug concentrations were affected by age, malnutrition and INH acetylator phenotype [16]. This study was planned to assess the plasma concentrations of isoniazid (INH), rifampicin (RIF), pyrazinamide (PZA) and ethambutol (EMB) achieved in children. The effect of different doses on the drug concentrations was evaluated for RIF and INH, only; while the effect of malnutrition was explored for all 4 drugs. An attempt was also made to correlate plasma concentrations of the said drugs with clinical outcome of therapy.

## Methods

This prospective study was carried out in a tertiary care hospital in New Delhi, India from 2009 to 2012. Children were screened and then enrolled from TB clinic services of the Department of Pediatrics, All India Institute of Medical Sciences, New Delhi. The study was approved by the Ethics Committee of the All India Institute of Medical Sciences. Written informed consent was taken from parents/guardians, assent was taken from children above seven years of age.

Children 6 months- 15 years age, who were newly diagnosed to have tuberculosis (pulmonary or extrapulmonary) and administered Category I ATT, were eligible for the study. Children with known HIV infection or any history, symptoms or signs suggestive of HIV infection, with poor general condition with high chances of mortality, known hepatic (transaminases > 5 times the upper normal limit) or renal dysfunction (creatinine > 0.7 mg/dL), adherence to ATT < 80% in two weeks preceding the pharmacokinetic session, those with chronic diarrhea/steatorrhea at the time of enrollment, and those with abdominal tuberculosis were excluded.

In the initial part of the study (2009–2011), children received the standard dosing schedule of ATT. Subsequently the second group was enrolled who received the revised dosing schedule of INH and RIF (Table 1). The national guidelines [17] were revised in favor of higher doses of isoniazid and rifampicin in 2012. However, even till date the same has not been operationalized. The enrollment in the standard dosing group was continued in view of the fact that data on Indian children generated from well-planned pharmacokinetic study were lacking and the findings of the current study would provide more robust evidence in support of these dose changes. Each of these groups had two subgroups: children without severe malnutrition and children with severe malnutrition. For the purpose of this study, severe malnutrition was defined in children upto five years of age as weight for height/length < 70% of expected [expected being 50^th^centile of CDC weight for height/length curve] and in children above five years as body mass index (BMI) for age < 5th percentile [18,19]. The definition of malnutrition that was used in clinical practice at the beginning of the study was used in the proposal and for sake of uniformity was not changed later on.

**Table 1:** **Recommended doses of first-line anti-TB drugs for children**

**Drugs**	**Standard dose (mg/kg), range (22)**	**Revised dose (mg/kg), range (22)**
**Isoniazid**	5 (4–6)	10 (7–15)
**Rifampicin**	10 (8–12)	15 (10–20)

Diagnosis of tuberculosis was established according to the standard guidelines [20]. Confirmation of *Mycobacterium tuberculosis* by Ziehl Neelsen staining and MGIT-960®culture system was attempted from any biological sample as feasible.

On enrollment a detailed history was taken, physical examination and anthropometric assessment (weight, height) were carried out.

### Treatment

Category based anti-tubercular therapy as recommended by Revised National Tuberculosis Control Program (RNTCP) was administered [21], daily therapy was followed - four drugs (isoniazid, rifampicin, pyrazinamide and ethambutol) in intensive phase for first two months followed by two drugs (isoniazid and rifampicin) for four months during maintenance phase. This is considered as category I as per RNTCP. For children continuing to have clinical symptoms or radiological findings attributable to tuberculosis at the end of 2 months, the intensive phase was extended for another one month. The maintenance phase was extended for 10 months in cases of osteo-articular TB.

In the first group, dosages used were INH: 5 (4-6) mg/kg, RIF: 10 (8–12) mg/kg, PZA: 30–35 mg/kg, EMB: 20–25 mg/kg (Table 1) [21,22]. Fixed dose combinations (Isoniazid-50 mg, Rifampicin-100 mg, Pyrazinamide- 300 mg per dispersible tablet) and ethambutol (200 mg) tablets were procured from Macleods Pharmaceuticals Limited, Mumbai, India.

In the second group, isoniazid and rifampicin were administered according to the revised dosage recommended by WHO- INH: 10 (7–15) mg/kg, RIF: 15 (10–20) mg/kg, PZA: 30-35 mg/kg, EMB: 20–25 mg/kg (Table 1) [12]. Fixed dose combinations (Isoniazid-75 mg, Rifampicin-100 mg, Pyrazinamide- 250 mg per dispersible tablet, Concept Pharmaceutical Limited, India) were used along with separate tablets for ethambutol (200 mg), same as the first group (see Additional file 1 for tables describing the weight band based dosing for both groups of children).

Depending on their ability, children either swallowed the tablets with minimal water or the dispersible medicines were dissolved in a teaspoon of water and administered under supervision of study personnel.

### Blood sampling

The samples for pharmacokinetic study were collected 14 days (upto 30 days) after starting ATT in all children. Pharmacokinetic sessions were planned such that the first sampling was done at least 24 hours from the last dose of ATT. On the day of scheduled blood sampling, enrolled children were asked to report in empty stomach in the morning to the pediatric services. The first blood sample (0 hour) was collected before administration of any drug. Immediately following the 0 hour sample, the medicines based on body weight measured at that point was administered. Next three point samplings at 1, 2 and 4 hours were done, calculating from the time of intake of drugs. Breakfast was allowed one hour after the drug, after collecting the blood sample at that point. At each time point, 0.5 mL blood was collected in polypropylene tube wrapped in aluminum foil, containing EDTA and sodium ascorbate and kept at 4°C. Plasma was separated soon after and stored at −80°C till further analysis. The same schedule was followed in both the dosage groups.

### Measurement of anti-tubercular drug concentrations in plasma by liquid chromatography-mass spectrometry (LC-MS/MS)

We used Ultra High Performance Liquid Chromatographic system (UHPLC, Thermo Surveyor system, CITY, USA) coupled with a triple quadrupole mass spectrometer, API-4000 QTrap equipped with Turbo Ion Spray ionization (ESI) source (MDS SCIEX, Applied Biosystems, USA), operated in positive mode for separation and detection of analytes. Standards for isoniazid, rifampicin, pyrazinamide, ethambutol and 6- amino-nicotinic acid (6-ANA) were procured from Sigma-Aldrich Ltd, India, having purity greater than 99%. Standard for acetyl isoniazid was synthesized in-house [23]. Linear calibration curves for isoniazid, rifampicin, pyrazinamide, ethambutol and acetyl isoniazid were prepared separately, keeping r > =0.99. The LLOD (lower limit of detection) and LLOQ (lower limit of quantification) for each of the analytes were: INH = 0.011 μg/mL and 0.014 μg/mL, RIF = 0.025 μg/mL and 0.031 μg/mL, PZA = 0.054 μg/mL and 0.547 μg/mL, ETB = 0.015 μg/mL and 0.018 μg/mL. Spiked sample calibration curve was subjected to inter-day and intraday variations’ analysis. The calculated coefficient of variation for their inter-day and intraday variation was <10%.

### Pharmacokinetic parameters

The pharmacokinetic parameters of C_max_ (maximum plasma concentration achieved) and T_max_ (time taken to achieve the maximum concentration) were determined by visual inspection of the data. Area under curve from 0–4 hours (AUC_0–4_) was calculated by the linear trapezoidal rule. The actual values for plasma concentrations at various time points as determined by LCMS/MS assay were considered for analysis.

The lower cut-offs for acceptable two hour plasma concentration or the usually targeted concentrations in adults were as follows: Isoniazid <3 μg/mL, rifampicin <8 μg/mL, pyrazinamide <20 μg/mL and ethambutol <2 μg/mL [24].

Children with acetyl-isoniazid/isoniazid ratio at 4 hours of less than 1.0 were considered as slow acetylators [25].

### Follow-up

The children were regularly followed-up till the completion of ATT and six months thereafter. Outcome of treatment was recorded in children for whom data for at least six months of ATT was available. Any child for whom change or extension of ATT regime was required or death occurred, was considered to be having a poor outcome. Good outcome was defined as completion of ATT within scheduled time. Any need for re-introduction of ATT after stopping therapy within the one year follow-up time period was defined as relapse. Category II ATT is defined as 2 months of INH + RIF+ EMB + PZA + injectable streptomycin, followed by one month of INH + RIF+ EMB + PZA, followed by 5 months of INH + RIF+ EMB [21].

### Sample size

Considering the inadequacy of data from well-designed studies in children in context to influence of malnutrition on pharmacokinetics of anti-tubercular drugs, a sample size of thirty children in each group of children was considered sufficient.

### Statistical analysis

Data were managed on MS Excel/Access softwares (version 2007) and analyzed using Stata 12.0 software (StataCorp, College Station, TX, USA). The significance of the differences in the categorical variables were assessed using chi-square or Fisher’s exact test and that of continuous variables using student’s *t* test/Wilcoxon rank-sum test as appropriate. A multivariate linear regression was done to predict the factors affecting 2 hour INH concentration, C_max_ and AUC_0–4_ of INH and RIF, with dose of INH and RIF in mg/kg, age, gender, weight for age and height for age z score as covariates. In case of INH, acetylator status was also included in the model.

A bivariate analysis (Wilcoxon ranksum test was used for the continuous variables, as the distribution was non-normal, whereas for categorical variables, chi-squared test was used) was carried out to identify the factors influencing the outcome of ATT in the enrolled children for whom follow-up data of at least six months were available. A multivariate logistic regression model was used to predict the factors affecting outcome with 2-hour plasma concentration of INH, RIF, PZA and EMB, age, weight for age and height for age z score and BCG vaccination status as covariates.

Weight-for-age (WAZ), height-for-age (HAZ) and weight-for-height (WHZ) Z scores and BMI z scores were calculated using the nutritional anthropometry module of EpiInfo version 5 (Centers for Disease Control and Prevention, Atlanta), based on the CDC-2000 curves [18].

## Results

We enrolled 127 children- 64 received the standard dose of ATT, whereas 63 received the revised dosage. The baseline characteristics of the enrolled children are outlined in Table 2. The children receiving standard or revised dosage of ATT were comparable in their baseline characteristics like age, gender, weight for height or BMI z-scores. All the children received category I ATT. Due to inadequate volume, one sample at 1 hour, 2 samples each at 2 and 4 hour time-points could not be assayed.

**Table 2:** **Baseline characteristics of children receiving standard and revised dosage of anti-tubercular therapy**

**Characteristics**	**Standard dosage, n = 64**		**Revised dosage, n = 63**	
	**Without severe malnutrition, n = 32**	**With severe malnutrition, n = 32**	**Without severe malnutrition, n = 37**	**With severe malnutrition, n = 26**
**Age, mean (SD), months**	97.2 (43.9)	105.9 (43.1)	126.5 (28.4)	91.3 (38.6)
**No of children, n (%)**				
**<2 years of age**	1 (3.1)	0	1 (2.7)	1 (3.8)
**2-5 years**	6 (18.8)	1 (3.1)	9 (24.3)	2 (7.7)
**>5 years**	25 (78.1)	31 (96.9)	27 (73)	23 (88.5)
**Boys, n (%)**	15 (46.9)	15 (46.9)	20 (54.1)	16 (61.5)
**Received BCG, n (%)**	27 (84.4)	21 (65.6)	24 (77.4)	20 (86.9)
**Weight for age z score, median (IQR)**	−1.5 (−2.1, -0.7)	−2.7 (−3.2, -1.8)	−1.3 (−1.7, -0.4)	−1.9 (−2.4, -1.4)
**Height for age z score, median (IQR)**	−1.4 (−2.03, 0)	−2.5 (−4.2, -1.6)	−1.1 (−1.9, -0.1)	−1.3 (−2.1, -0.7)
**Weight for height z score, median (IQR)**	−1.1 (−1.9, -0.7)	−1.5 (−1.9, -1.03)	−0.6 (−1.1, -0.2)	−1.9 (−2.2, -1.3)
**BMI z score, median (IQR)**	−0.8 (−1.4, -0.2)	−1.7 (−2.5, −0.6)	−0.7 (−1.2, −0.4)	−2.1 (−2.8, -1.8)
**AFB/MGIT positive, n (%)**	5 (15.6)	9 (28.1)	6 (16.2)	7 (26.9)
**Diagnosis, n (%)**				
**Progressive pulmonary disease**	18 (56.2)	18 (56.2)	11(29.7)	16 (61.5)
**Pleural effusion**	4 (12.5)	4 (12.5)	2 (5.4)	0
**Disseminated**	10 (31.3)	10 (31.3)	3 (8.1)	0
**Osteoarticular**	0	0	8 (21.6)	3(11.5)
**Peripheral lymphadenopathy**	0	0	13 (35.2)	7 (27)

IQR: interquartile range, SD: standard deviation, AFB: acid fast bacillus, MGIT: Mycobacterial growth indicator tube, BCG: Bacillus Calmette Guerin.

### Isoniazid

The median C_max_ and 2-hour levels, which were below usually targeted concentrations with the initial doses, increased significantly after increasing the dose. The number of children with low 2-hour plasma concentrations was 59 (95.2%) for the standard dose group as compared to 43 (68.3%) for the revised dose group, p < 0.001 (Table 3). In the group with revised higher dose, the median C_max_ achieved was significantly higher in children <5 years of age [4.8 (3.8, 5.8) vs. 3.04 (1.6, 4.6) μg/mL, p = 0.03], whereas no such age wise difference could be ascertained at a dose of 5 mg/kg. The mean (SD) dose of INH in children younger than 5 years in the revised doses group was 12.1 (0.8) mg/kg and 11.2 (0.7) mg/kg for older children.

**Table 3:** **Pharmacokinetic parameters of isoniazid and rifampicin at standard and revised dosage**

**Parameter**	**Isoniazid**			**Rifampicin**		
	**Standard dose, n = 64**	**Revised dose, n = 63**	**p value**	**Standard dose, n = 64**	**Revised dose, n = 63**	**p value**
**Dose, mg/kg, mean (SD)**	5.6 (0.5)	11.4 (0.8)	<0.0001	11.2 (1.0)	15.2 (1.1)	<0.0001
**2-hour concentration,** **μg/mL**	0.4 (0.2, 0.8)	2.0 (1.2, 3.7)	<0.0001	8.8 (5.4, 11.9)	9.9 (5.4, 19.9)	0.06
**C**_**max**_ **,** **μg/mL**	0.7 (0.3, 1.2)	3.4 (1.8, 5.0)	<0.0001	10.4 (7.2, 13.9)	12.0 (6.1, 24.3)	0.08
**T**_**max**_ **, hour**	1 (1, 2)	1 (1, 2)	0.6	1.5 (1, 2)	2 (2, 3)	0.004
**AUC**_**0–4**_ **,** μg/mL*hr	1.5 (0.8, 2.6)	7.0 (4.4, 11.9)	<0.0001	28.0 (19.3, 35.5)	30.5 (17.1, 58.9)	0.2
Low 2-hr concentration*, n (%)	59 (95.2)	43 (68.3)	<0.001	27 (43.5)	26 (41.3)	0.79

Values are expressed as median (IQR) unless specified.

*Low 2-hr concentrations: plasma concentration of isoniazid <3 μg/mL, rifampicin <8 μg/mL at 2 hour post drug time point.

C_max_: maximum concentration, T_max_: Time needed to reach the maximum concentration, AUC: area under curve.

### Acetylator status

The acetylator phenotype was determined in 122 children. Fast acetylator phenotype was ascertained in 81.2% of the children, whereas 18.8% were slow acetylators. In the group of children receiving standard dosage, 86.7% (52 children) were fast acetylators and 13.3% (8 children) were slow acetylators; amongst the severely malnourished children in this group 90% (27 children) were fast acetylators, whereas amongst the children who were not severely malnourished 83.3% were fast acetylators. In the other group receiving revised dosage, 75.8% (47 children) were fast acetylators whereas 24.2% (15 children) were slow acetylators; percentages of fast acetylators amongst children with or without severe malnutrition in this group were 84.6% and 69.4%, respectively. In the first group, the median 2-hour plasma concentration [1.2 (0.6, 2.9) *vs.* 0.4 (0.1, 0.7) μg/mL, p = 0.001] as well as C_max_ achieved [1.8 (1.4, 3.4) *vs.* 0.6 (0.3, 0.9) μg/mL, p = 0.0001] were higher in slow acetylators as compared to the fast acetylators. AUC_0–4_ obtained was also higher in the slow acetylators [4.5 (3.2, 9.7) *vs.* 1.2 (0.7, 2.1) μg/mL*hr, p = 0.0001]. Similarly in the revised doses group, slow acetylators attained a higher 2-hour concentration [3.8 (2.7, 5.1) vs. 1.6 (1.1, 2.9), p = 0.003] and C_max_ [4.6 (3.4, 7.2) vs. 2.9 (1.5, 4.7), p = 0.007] of INH as compared to the fast acetylators.

When a multivariate linear regression was done, only lower dose of INH in mg/kg and fast acetylator status were significantly associated with lower 2-hour INH concentration, C_max_ and AUC_0–4_ after adjusting for age, gender, weight for age and height for age z score.

### Rifampicin

The median concentration reached at two hour time-point and C_max_ increased marginally with the increase in dose, the changes were not statistically significant. AUC_0–4_ also increased marginally (p = 0.2). Number of children with low 2-hour plasma concentration remained almost unaltered (43.5% vs. 41.3%, p = 0.8) (Table 3). There was no influence of age on the 2-hour concentration of RIF at either of the doses. A linear regression showed that lower 2-hour RIF concentrations, C_max_ and AUC_0–4_ were significantly associated with only dose in mg/kg of RIF when adjusted for age, gender, weight for age and height for age z score.

### Pyrazinamide

Dose of pyrazinamide above 30 mg/kg (mean: 35.8, SD: 3.6) resulted in plasma concentrations of the drug above the acceptable cut-off levels. Only eight (6.4%) children had low 2-hour plasma levels (Table 4).

**Table 4:** **Pharmacokinetic parameters of ethambutol and pyrazinamide in 127 children**

**Parameter**	**Ethambutol**	**Pyrazinamide**
**Dose, mg/kg, mean (SD)**	21.7 (2.4)	35.7 (3.6)
**2-hour concentration,** **μg/mL**	1.5 (0.6, 3.1)	42.7 (34.9, 52.9)
**C**_**max**_ **,** **μg/mL**	2.1 (1.0, 3.7)	47.8 (39.4, 58.5)
**T**_**max**_ **, hours**	2 (1, 2)	2 (1, 2)
**AUC**_**0–4**_ **,** μg/mL*hr	4.8 (2.2, 8.9)	140.5 (117.4, 173.2)
Low 2-hr concentration*, n (%)	71 (55.9)	8 (6.4)

Values are expressed as median (IQR) unless specified.

*Low 2-hr concentration: plasma concentration of ethambutol <2 μg/mL, pyrazinamide <20 μg/mL at 2 hour post drug time point.

C_max_: maximum concentration, T_max_: Time needed to reach the maximum concentration, AUC: area under curve.

### Ethambutol

More than half (55.9%) of the children had 2-hour concentration lower than the usually targeted levels. Details are outlined in Table 4.

In the first group, 2-hour plasma concentrations of at least one drug were low in 59 (92.2%) children. With the revised doses, 54 (85.7%) children had low 2-hour plasma concentrations of at least one drug.

### Effect of nutritional status on pharmacokinetics of isoniazid, rifampicin, pyrazinamide and ethambutol

The pharmacokinetic parameters of isoniazid and rifampicin in either of the dosing schedules and that of pyrazinamide or ethambutol (C_max_, AUC_0–4_, T_max_) and number of children with low two hour plasma concentration did not differ in children with or without severe malnutrition (see Additional file 1).

### Treatment response

Evaluation of treatment response at the end of six months of anti-tubercular therapy were available in 62 children of the first group and 61 children in the second group receiving revised dosage of ATT.

In the first group, category II ATT had to be started due to non-resolution of symptoms or radiological findings in 4 (6.2%) children, 2nd line ATT was started in one (1.6%) child. One (1.6%) child had relapse of disease after completion of therapy and was administered category II treatment. Hepatitis developed in 2 (3.2%) children. With the standard dosing schedule, nature of outcome was not associated with the plasma concentrations or AUC_0–4_ achieved for any of the four drugs studied. However, there was a trend of median (IQR) C_max_ of INH being lower in children with poor outcome [1.3 (0.7, 1.5) vs. 3.4 (1.8, 5.0) μg/mL, p = 0.05].

Only confirmation of *Mycobacterium tuberculosis* was associated with the outcome, with children with poor outcome having a higher percentage of confirmed cases (55.6% *vs*. 16.4%, p = 0.01) (Table 5). A multivariate logistic regression model showed that there was no association of 2-hour plasma concentration of INH, RIF, PZA and EMB with poor outcome, when adjusted for age, weight for age and height for age z score and BCG vaccination status.

**Table 5:** **Factors affecting outcome of therapy in children with at least six months of follow-up on ATT**

	**Standard dosage of ATT, n = 62**			**Revised dosage of ATT, n = 61**		
**Variables**	**Good outcome n = 53**	**Poor outcome n = 9**	**p value**	**Good outcome n = 44**	**Poor outcome n = 17**	**p value**
**2-hr plasma isoniazid concentration,** **μg/mL**	0.4 (0.2, 0.8)	0.5 (0.2, 0.8)	0.9	2.3 (1.2, 3.9)	1.7 (1.3, 2.8)	0.2
**2-hr plasma rifampicin concentration,** **μg/mL**	8.7 (5.4, 11.9)	9.7 (3.9, 11.6)	0.8	9.5 (5.3, 21.2)	11.3 (5.7, 15.2)	0.9
**2-hr pyrazinamide concentration,** **μg/mL**	40.4 (34.8, 46.9)	44.2 (38.4, 53.1)	0.7	39.8 (35.0, 44.7)	41.4 (34.4, 47.8)	0.8
**2-hr ethambutol concentration,** **μg/mL**	0.7 (0.4, 1.5)	0.6 (0.5, 0.9)	0.4	2.5 (1.7, 3.7)	3.2 (2.2, 4.8)	0.2
**C**_**max**_ **isoniazid,** **μg/mL**	3.4 (1.8, 5.0)	1.3 (0.7, 1.5)	0.05	3.2 (2.0, 4.7)	3.5 (1.8, 5.2)	0.6
**C**_**max**_ **rifampicin,** **μg/mL**	10.3 (6.8, 13.9)	11.5 (7.8, 14.3)	0.9	10.4 (7.7, 14.0)	12.8 (6.6, 15.3)	0.5
**C**_**max**_ **pyrazinamide,** **μg/mL**	50.1 (39.2, 67.5)	52.8 (43.5, 68.5)	0.8	46.7 (40.4, 52.6)	43.4 (38.4, 53.9)	0.5
**C**_**max**_ **ethambutol,** **μg/mL**	1.1 (0.7, 2.1)	1.2 (0.5, 1.9)	0.8	3.3 (2.3, 4.1)	3.7 (2.3, 4.9)	0.4
**AUC**_**0–4**_ **isoniazid,** μg/mL*hr	1.3 (0.7, 2.4)	1.9 (1.5, 3.3)	0.2	7.9 (4.2, 13.2)	5.9 (4.5, 10.1)	0.3
**AUC**_**0–4**_ **rifampicin,** μg/mL*hr	27.9 (19.1, 36.1)	30.2 (23.5, 34.1)	0.8	25.3 (16.6, 60.7)	30.6 (21.7, 44.1)	0.8
**AUC**_**0–4**_ **pyrazinamide,** μg/mL*hr	159.9 (111.8, 207.0)	155.3 (125.3, 210.9)	0.9	137.8 (123.6, 158.3)	133.7 (121.4, 151.6)	0.5
**AUC**_**0–4**_ **ethambutol,** μg/mL*hr	2.5 (1.5, 5.5)	2.1 (1.4, 3.7)	0.5	7.7 (4.9, 10.7)	10.0 (5.9, 10.9)	0.5
**WAZ at baseline**	−1.8 (−2.8, -1.1)	−1.9 (−3.2, -1.7)	0.3	−1.3(−1.9, -0.6)	−1.9 (−2.3, -1.8)	0.007
**HAZ at baseline**	−1.5 (−2.1, -0.2)	−1.2 (−1.9, -0.2)	0.8	−1.1 (−2.3, -0.2)	−1.5 (−1.9, -0.8)	0.5
**Received BCG, n (%)**	42 (76.4)	6 (66.7)	0.7	31 (70.4)	11 (64.7)	0.7
**Mycobacterium confirmed, n (%)**	9 (16.4)	5 (55.6)	0.01	8 (18.6)	4 (23.5)	0.7
**Diagnosis, n (%)**			0.9			0.5
**Progressive pulmonary disease**	30 (56.6)	5 (55.6)		17 (38.6)	10 (58.8)	
**Pleural effusion**	7 (13.2)	1 (11.1)		1 (2.3)	1 (5.9)	
**Disseminated**	16 (30.2)	3 (33.3)		2 (4.5)	1 (5.9)	
**Osteoarticular**	0	0		9 (20.5)	2 (11.8)	
**Peripheral lymphadenopathy**	0	0		15 (34.1)	3 (17.6)	

Values are median (IQR) unless specified, AUC: area under curve, WAZ: weight for age z score, HAZ: height for age z score, BCG: Bacillus Calmette–Guérin, IQR: interquartile range, C_max_: maximum concentration.

In the second group, three (4.9%) children were administered category II ATT due to worsening or non-improvement of symptoms, one (1.6%) child had relapse of disease. There was only one death in a child with disseminated TB and severe malnutrition. Three (4.9%) children had developed features of hepatitis in this group. Even with the increased dosage schedule, concentrations of the four drugs studied were not associated with the outcome of treatment. In this group, children with lower weight and BMI z score had poorer outcome (Table 5).

## Discussion

The current study, conducted in 127 children with tuberculosis, observed that most of the children had low levels of at least one of the four drugs, the plasma levels of isoniazid and ethambutol were suboptimal in majority of children enrolled. With the revised dosage, concentrations of both isoniazid and rifampicin increased, though the change was not statistically significant in case of rifampicin. We did not observe an association between low plasma drug concentrations and poor outcome. There was no effect of malnutrition on 2-hour concentrations, C_max_, T_max_, and AUC_0–4_ of isoniazid, rifampicin, pyrazinamide and ethambutol in either of the two dosing schedules used.

Multiple time point sampling as in conventional pharmacokinetic studies is challenging in children as there are concerns regarding parental consent, ethical issues and collection of samples. A limited sampling approach at four time points was adopted in this study. This strategy had been validated for rifampicin in an earlier study from the same institute conducted in adults to study the absorption phase [26]. Though it was not possible to comment on parameters like half-life and volume of clearance, the absorption spectrum could be well studied.

The assays used for measurement of serum or plasma drug concentrations in the previous studies on pharmacokinetics of anti-tubercular drugs in children have been varied and hence, the comparison of results obtained is challenging. Of various assays to measure the plasma drug levels, LC-MS/MS is the most sophisticated and sensitive, only a few studies in children with tuberculosis have used this assay [7,8]. In the current study, the plasma concentrations of all the four first line anti-tubercular drugs were measured simultaneously by LC-MS/MS. This allowed a high throughput and sensitive assay.

The plasma levels of the anti-tubercular drugs have been evaluated both for intermittent dosing, [10,16,27,28] and daily schedules [7,8,29-31]. There are differences in the formulations used in various studies. Some of the studies used individual, separate tablets for each drug studied, [16,30-32] while in many other studies fixed dose formulations of isoniazid, rifampicin and pyrazinamide were used [7-9,29,33]. The current study reports the pharmacokinetic details of isoniazid, rifampicin, pyrazinamide as well as ethambutol in Indian children, after daily dosing of the medicines using fixed dose formulations of isoniazid, rifampicin and pyrazinamide along with separate tablets of ethambutol.

### Isoniazid

Several studies have reported low 2-hour concentration or low C_max_ of isoniazid in large proportion of children with doses of around 5 mg/kg/day, though not in the proportions observed in the current study. In earlier studies up to 83% children have been reported to have a suboptimal plasma concentration of isoniazid. [8,29] While some authors have reported low levels in only 12-14% of children studied [16,34].

Thee et al. reported low C_max_ in half of the children following a dose of 5 mg/kg of isoniazid, but in none after dose of 10 mg/kg in children younger than two years [33]. Recent simulation studies have showed that higher doses lead to increased C_max_ and isoniazid exposure [35,36]. A study on 20 low birth weight infants demonstrated that though adequate C_max_ was achieved at a dose of 10 mg/kg INH, elimination was reduced in younger and smaller infants, thus raising a note of caution against using higher doses in this age group [37]. Our study showed a significantly higher increase in C_max_ and AUC_0–4_ in younger children (less than five years), when the revised doses were used. Hence the impact of increasing the dose may be different in children according to age groups and should be taken into account while hiking the INH doses.

Acetylator status predicted the isoniazid exposure in our cohort of children, fast acetylators having a lower 2-hour level, C_max_ and AUC_0–4_. This corroborates with the findings of other researchers who also observed an association of lower isoniazid concentrations with fast acetylator phenotype [6,8]. Acetylation status varies according to the ethnicity of populations. India has a population of diverse gene pool varying with geographical regions [38]. There have been dissimilar reports from different parts of India on the acetylator status of Indian population, determined phenotypically and genotypically. Studies from south India have established a predominance of slow acetylators up to 74% of the population [39]. A recent study by Ramachandran found 32% fast acetylators in south Indian children, determined by estimation of salivary INH levels [16]. However other studies from north Indian adult population have reported lower proportion of slow acetylators, [40-42] ranging from 14.5% to 35.4%. The distribution of acetylator phenotype in the current study, with 81.2% fast acetylators, matches that of north Indian population. Since acetylator status is an important factor in the plasma concentration of INH achieved by a person, intra and inter population differences will have an impact on determining the doses needed for a particular population and the guidelines issued by any program.

Increasing the dose has significantly improved the plasma concentrations in our study, providing supportive evidence to the revised dosing schedule of WHO. But the number of children with low two hour levels still remain unacceptably high (68.2%), factors other than just increasing the dose must be sought to explain this. Also some caution and more studies may be warranted while using the higher dose in younger children, as shown in our study that younger children may achieve better plasma concentrations of the drug. Recent WHO guidelines have recommended isoniazid in the range of 7–15 mg/kg mainly to facilitate ease of administration with the currently available FDCs [43].

### Rifampicin

Several studies have raised concern regarding inadequate exposure of rifampicin after a dose of around 10 mg/kg. Seth et al. demonstrated a C_max_ of 3.3- 3.8 μg/mL after an average dose of 12 mg/kg of rifampicin [44]. In recent studies with doses of around 10 mg/kg up to 90% children had sub-therapeutic maximum concentrations of rifampicin [7,16,29].

Using the revised WHO dosing schedule, Thee et al. demonstrated that C_max_ and AUC were significantly augmented when dose of rifampicin was increased from 10 to 15 mg/kg. A dose of 15 mg/kg led to only three out of eleven children having suboptimal peak concentration of rifampicin [33]. In our study the concentrations of rifampicin did increase with dose, but not significantly, neither did the number of children with low two hour levels vary. Inspite of initial concerns, studies have now documented that the bioavailability from fixed dose combinations and single, separate formulations are equivalent [45]. Hence, the use of fixed dose combinations in our study, may not have contributed substantially to the low levels of concerned drugs. Again there may be other factors like malabsorption, drug-interactions, efflux proteins, contributing to the low levels other than just inadequate dose.

### Pyrazinamide

There have been few reports in children regarding low levels of pyrazinamide. Approximately one-thirds of children receiving 30–33 mg/kg of pyrazinamide have been reported to have low peak concentrations [9,10,16,29,30,46].

Our study demonstrated that concentrations achieved by administering pyrazinamide above 30 mg/kg were adequate and very few children had inadequate 2-hour levels of pyrazinamide.

### Ethambutol

Data regarding pharmacokinetics of ethambutol in children is limited globally. Various authors have reported low levels of plasma ethambutol concentrations [10,47,48], the average doses ranged from 15 mg/kg to 25 mg/kg. In view of more than half (55.9%) of the children in our study having low 2-hour levels of ethambutol at a mean dose of 21.7 mg/kg, consideration may be given to increasing the dose, albeit keeping in mind the possible toxicities. However, additional pharmacokinetic studies are needed to garner further evidence before any suggestions can be made.

### Effect of malnutrition

In our study, we did not observe any effect of malnutrition on 2 hour concentrations, C_max_, T_max_, and AUC_0–4_ of isoniazid, rifampicin, pyrazinamide or ethambutol, even with enhanced dosing. There are contrasting reports of influence of nutritional status on exposure of anti-tubercular drugs in children. Some earlier studies on isoniazid and rifampicin reported that serum concentrations of these drugs were higher in malnourished children [49].

Other studies in children with poor nutritional status have suggested that bioavailability of anti-tubercular drugs, especially rifampicin and pyrazinamide is lower in malnutrition [16,50]. On the other hand, similar to our results, few studies have reported absence of effect of malnutrition on pharmacokinetics of anti- tubercular drug levels [8,10,27,33,51,52].

There are various pathophysiological changes associated with malnutrition which may affect different aspects of drug pharmacokinetics, it is difficult to predict the result of interplay of the various effects. This study and the previous studies have estimated the total plasma concentrations; it may be worthwhile to determine the free drug concentrations in plasma as well as the intracellular drug concentrations, the latter may particularly be relevant as the pathogen is intracellular.

### Correlation with treatment response

The outcome of tuberculosis in this cohort of children was not influenced by the levels of anti-tubercular drugs studied. However, there was a trend of median C_max_ of INH being lower in children with poor outcome at the dose of 5 mg/kg. The cut off levels for optimal plasma concentrations of anti-tubercular drugs have been derived from adult healthy volunteers studied under controlled environment and validated at National Jewish Medical and Research Center by Peloquin et al. [24]. These two hour cut-off values have since been widely used worldwide by various researchers. But the optimal concentrations required for effective therapy and hence, cure of tuberculosis in children are not exactly known as there are very few studies which have tried to associate dose and serum concentrations of anti-tubercular drugs with successful outcome of therapy.

In a study on Indian children on intermittent ATT, unfavorable treatment outcome was found to be associated with lower isoniazid and rifampicin exposure [16]. A South African study primarily designed to assess the difference in serum concentration of rifampicin in HIV infected and uninfected children, demonstrated no association between weight gain or chest X-ray clearance and any of the pharmacokinetic parameters of rifampicin [7]. In addition to the drug concentrations, the body’s immune system is likely to play a major role in tackling the tuberculosis disease. Also the drugs have to target the mycobacteria, most of which are intracellular, it may be important to study the intracellular drug concentrations as well. Synergistic action of pyrazinamide with rifampicin, isoniazid with ethambutol may compensate for the low levels of one drug [53]. This may explain the observation of acceptable outcomes in a scenario where most of the anti-tubercular drugs showed low levels of anti-tubercular drugs. However, there may be an effect on long-term likelihoods of relapse; this aspect has not been studied.

Interestingly, proportion of unfavourable outcome was higher in children receiving the increased doses of ATT. This group had higher numbers of children with extra-pulmonary disease and had lower weight-for-age Z scores than the group receiving lower doses; these may have affected the response to therapy.

Strengths of this study include the use of a highly sensitive assay for simultaneously estimating the plasma concentrations of all 4 first line anti-tubercular drugs in children. We have assessed the drug levels after modifying the doses of isoniazid and rifampicin. The present study also described the pharmacokinetics of ethambutol in Indian children for which data are not available in published literature. Also the revised dosages of isoniazid and rifampicin have been evaluated in the Indian children for the first time, enabling us to assess the efficacy of this regimen. We have also reported outcomes in the enrolled children.

The limitations of the study include the inability to evaluate pharmacokinetic details like half-life, rate of clearance, due to the truncated sampling strategy followed and use of 2-hour plasma concentration as a measure of optimal dose. The limited numbers of children in each of the treatment sub-groups complicates the assessment of association of outcome with plasma concentrations of the drugs.

## Conclusion

The results of this study have led credence to the concern that doses used in children were inadequate leading to inadequate plasma concentrations of isoniazid, rifampicin and ethambutol. Increasing the dose of isoniazid seems to have beneficial effects on plasma concentration achieved. But augmenting the dose of rifampicin has failed to significantly alter the concentrations achieved or number of children with low two hour levels. Malnutrition was not associated with low plasma concentrations of any of these drugs. No relationship was found between drug concentration and treatment response, even in augmented doses. We may have to be cautious while increasing the doses and strive to asses any other factors which may be influencing the drug concentrations and treatment outcomes in children.

## Additional file

Additional file 1:
**Table S1:** Weight band based dosage of fixed dose combination of isoniazid, rifampicin and pyrazinamide and ethambutol tablets in the first group of standard dosage schedule. **Table S2:** Weight band based dosage of fixed dose combination of isoniazid, rifampicin and pyrazinamide and ethambutol tablets in the second group of revised dosage schedule. **Table S3:** Effect of malnutrition on the pharmacokinetic parameters of isoniazid at different dosages. **Table S4:** Effect of malnutrition on the pharmacokinetic parameters of rifampicin at different dosages. **Table S5:** Effect of malnutrition on the pharmacokinetic parameters of pyrazinamide and ethambutol.

## Abbreviations

ATT: Anti-tubercular therapy
BMI: Body mass index
EMB: Ethambutol
HAZ: Height for age z score
INH: Isoniazid
LC-MS/MS: Liquid chromatography-mass spectrometry
PZA: Pyrazinamide
RIF: Rifampicin
RNTCP: Revised National Tuberculosis Control Program
TB: Tuberculosis
WAZ: Weight for age z score
WHO: World Health Organization
WHZ: Weight for age z score
