# Supplementary material for: Pharmacokinetics of isoniazid, rifampicin, pyrazinamide and ethambutol in Indian children
Source: BMC Infect Dis. 2015 Mar 14;15:126. doi: 10.1186/s12879-015-0862-7 (PMC4373095; doi:10.1186/s12879-015-0862-7)
Supplement: Additional file 1: — Table S1: Weight band based dosage of fixed dose combination of isoniazid, rifampicin and pyrazinamide and ethambutol tablets in the first group of standard dosage schedule. Table S2: Weight band based dosage of fixed dose combination of isoniazid, rifampicin and pyrazinamide and ethambutol tablets in the second group of revised dosage schedule. Table S3: Effect of malnutrition on the pharmacokinetic parameters of isoniazid at different dosages. Table S4: Effect of malnutrition on the pharmacokinetic parameters of rifampicin at different dosages. Table S5: Effect of malnutrition on the pharmacokinetic parameters of pyrazinamide and ethambutol. [file 12879_2015_862_MOESM1_ESM.docx]

**Additional file 1**

**Table S1: Weight band based dosage of fixed dose combination of isoniazid, rifampicin and pyrazinamide and ethambutol tablets in the first group of standard dosage schedule**

| **Body weight**  **(kg)** | **FDC (Isoniazid-50mg, Rifampicin-100mg, Pyrazinamide -300mg)** | **Ethambutol (200 mg)** |
| --- | --- | --- |
| < 5 | 0.5 | 0.5 |
| 5-10 | 1 | 1 |
| 11-15 | 1.5 | 1.5 |
| 16-20 | 2 | 2 |
| 21-25 | 2.5 | 2.5 |
| 26-30 | 3 | 3 |
| 31-35 | 3.5 | 3.5 |
| 36-40 | 4 | 4 |
| 41-45 | 4.5 | 4.5 |
| 46-50 | 5 | 5 |

FDC: fixed dose combination

**Table S2: Weight band based dosage of fixed dose combination of isoniazid, rifampicin and pyrazinamide and ethambutol tablets in the second group of reviseddosage schedule**

| **Body weight**  **(kg)** | **FDC (Isoniazid-75mg, Rifampicin-100mg, Pyrazinamide -250mg)** | **Ethambutol (200 mg)** |
| --- | --- | --- |
| 6-7 | 1 | 0.75 |
| 7.1-8 | 1.25 | 0.75 |
| 8.1-9 | 1.5 | 0.75 |
| 9.1-11 | 1.5 | 1 |
| 11.1-16 | 2 | 1.5 |
| 16.1-18 | 2.5 | 2 |
| 18.1-23 | 3 | 2 |
| 23.1-27 | 4 | 2.5 |
| 27.1-29 | 4 | 3 |
| 29.1-32 | 4.5 | 3 |
| 32.1-35 | 5 | 3 |

FDC: fixed dose combination

**Table S3: Effect of malnutrition on the pharmacokinetic parameters of isoniazid at different dosages**

|  | Standard dose | | p value | Revised dose | | p value |
| --- | --- | --- | --- | --- | --- | --- |
|  | Children with severe malnutrition, n=32 | Children without severe malnutrition, n=32 |  | Children with severe malnutrition, n= 37 | Children without severe malnutition, n= 26 |  |
| **Dose, mean (SD), mg/kg** | 5.7 (0.6) | 5.5 (0.4) | 0.2 | 11.4 (0.8) | 11.4 (0.8) | 0.9 |
| **2-hour concentration, µg/mL** | 0.4 (0.1,0.8) | 0.5 (0.2,0.9) | 0.7 | 1.7 (1.3,3.0) | 2.6 (1.2,4.0) | 0.4 |
| **C_max_, µg/mL** | 0.7 (0.3, 1.0) | 0.7 (0.4, 1.3) | 0.5 | 2.9(1.6,3.9) | 3.9 (2.1,5.8) | 0.5 |
| **T_max_** | 1 (1,2) | 1 (1,2) | 0.7 | 1 (1,2) | 1 (1,1) | 0.2 |
| **AUC_0-4_, µg/mL*hr** | 1.2 (0.7, 2.1) | 1.7 (0.9, 2.9) | 0.6 | 6.2 (4.5,9.9) | 8.3 (4.4,13.2) | 0.3 |
| **Low 2-hr concentration*, n (%)** | 29/31 (96.8) | 30/31 (93.5) | 0.3 | 19(73.1) | 24 (64.9) | 0.1 |

Values are expressed as median (IQR) unless specified.

*Low 2-hr concentration = plasma concentration of isoniazid <3 µg/mL at two hour time point

C_max_: maximum concentration, T_max_: Time needed to reach the maximum concentration, AUC: area under concentration

**Table S4: Effect of malnutrition on the pharmacokinetic parameters of rifampicin at different dosages**

|  | Standard dose | | p value | Revised dose | | p value |
| --- | --- | --- | --- | --- | --- | --- |
|  | Children with severe malnutrition, n=32 | Children without severe malnutrition, n=32 |  | Children with severe malnutrition, n= 26 | Children without severe malnutition, n= 37 |  |
| **Dose, mean (SD), mg/kg** | 11.3 (1.2) | 11.0 (0.8) | 0.2 | 15.2 (1.0) | 15.2 (1.1) | 0.9 |
| **2-hour concentration, µg/mL** | 8.5 (5.6, 12.0) | 8.9(4.7, 10.7) | 0.8 | 11.6 (4.3,23.3) | 9.8 (5.7,17.0) | 0.4 |
| **C_max_, µg/mL** | 10.1(6.3,13.8) | 11.3 (8.1,14.3) | 0.4 | 13.2 (5.3,25.7) | 11.3 (8.1, 23.6) | 0.5 |
| **T_max_** | 2 (1,2) | 1 (1,1) | 0.5 | 2 (2,3) | 2 (2,3) | 0.2 |
| **AUC_0-4_, µg/mL*hr** | 28.4(17.7,35.1) | 27.9 (20.1,37.5) | 0.8 | 32.8(11.7,66.7) | 26.3(21.0,47.4) | 0.3 |
| **Low 2-hr concentration*, n (%)** | 11(35.5) | 11(35.5) | 0.6 | 12 (37.8) | 14(46.1) | 0.1 |

Values are expressed as median (IQR) unless specified.

* Low 2-hr concentration = plasma concentration of rifampicin <8 µg/mL

C_max_: maximum concentration, T_max_: Time needed to reach the maximum concentration, AUC: area under concentration

**Table S5: Effect of malnutrition on the pharmacokinetic parameters of pyrazinamide and ethambutol**

|  | Pyrazinamide | | p value | Ethambutol | | p value |
| --- | --- | --- | --- | --- | --- | --- |
|  | Children with severe malnutrition, n=58 | Children without severe malnutition, n=69 |  | Children with severe malnutrition, n= 58 | Children without severe malnutition, n= 69 |  |
| **Dose, mean (SD), mg/kg** | 35.3 (3.5) | 36.2 (3.6) | 0.2 | 21.6 (2.2) | 21.8 (2.6) | 0.6 |
| **2-hour concentration, µg/mL** | 41.9 (36.3,53.0) | 44.2 (34.4,52.7) | 0.9 | 1.5 (0.7,2.9) | 1.7 (0.5,3.3) | 0.8 |
| **C_max_, µg/mL** | 46.9 (39.8,60.1) | 48.7 (39.1,58.4) | 0.9 | 2.0(1.0,3.4) | 2.3(0.9,4.1) | 0.5 |
| **T_max_** | 2(1,2) | 1 (1,2) | 0.4 | 2 (1,2) | 2 (1,2) | 0.3 |
| **AUC_0-4_, µg/mL*hr** | 133.8 (115.6,164.6) | 143.8(122.7,174.1) | 0.3 | 4.3 (2.3,6.4) | 56(2.2,10.3) | 0.2 |
| **Low 2-hr concentration*, n (%)** | 3 (5.3) | 5 (7.3) | 0.6 | 33 (56.9) | 36(52.2) | 0.6 |

Values are expressed as median (IQR) unless specified.

* Low 2-hr concentration = plasma concentration of rifampicin <8 µg/mL

C_max_: maximum concentration, T_max_: Time needed to reach the maximum concentration, AUC: area under concentration
